# Supplementary material for: Fast TILs—A pipeline for efficient TILs estimation in non-small cell Lung cancer
Source: J Pathol Inform. 2025 Mar 12;17:100437. doi: 10.1016/j.jpi.2025.100437 (PMC11994347; doi:10.1016/j.jpi.2025.100437)
Supplement: Supplementary file 1 — Supplementary information [file mmc1.docx]

# Appendix

## Supplementary Methods

### Definition of metrics used to evaluate the performance of the cell segmentation model

The results of our cell segmentation pipeline are detailed in Shvetsov et al 2022. Briefly, we use the following definitions of metrics to evaluate the cell segmentation pipeline:

1. $DICE=\frac{2*TP}{2*TP+FP+FN}$ , where *TP*, *FP* and *FN* represent the number of true positive, false positive and false negative pixels, respectively.
2. $\text{IoU}=\frac{\sum_{i=1}^{n} \left| X_{i}\cap Y_{i} \right|}{\sum_{i=1}^{n} \left| X_{i}\cup Y_{i} \right|}$ , where n represents the number of classes, where *X_i_* represents the set of pixels predicted to belong to class *i*, and *Y_i_* represents the set of pixels that belong to class *i* in the ground truth.
3. $PQ= \frac{IOU}{TP+0.5FP+0.5FN}$ , where *TP*, *FP* and *FN* represent the number of true positives, false positive and false negative pixels, respectively, and the *IoU* score is defined as described in Equation 2).


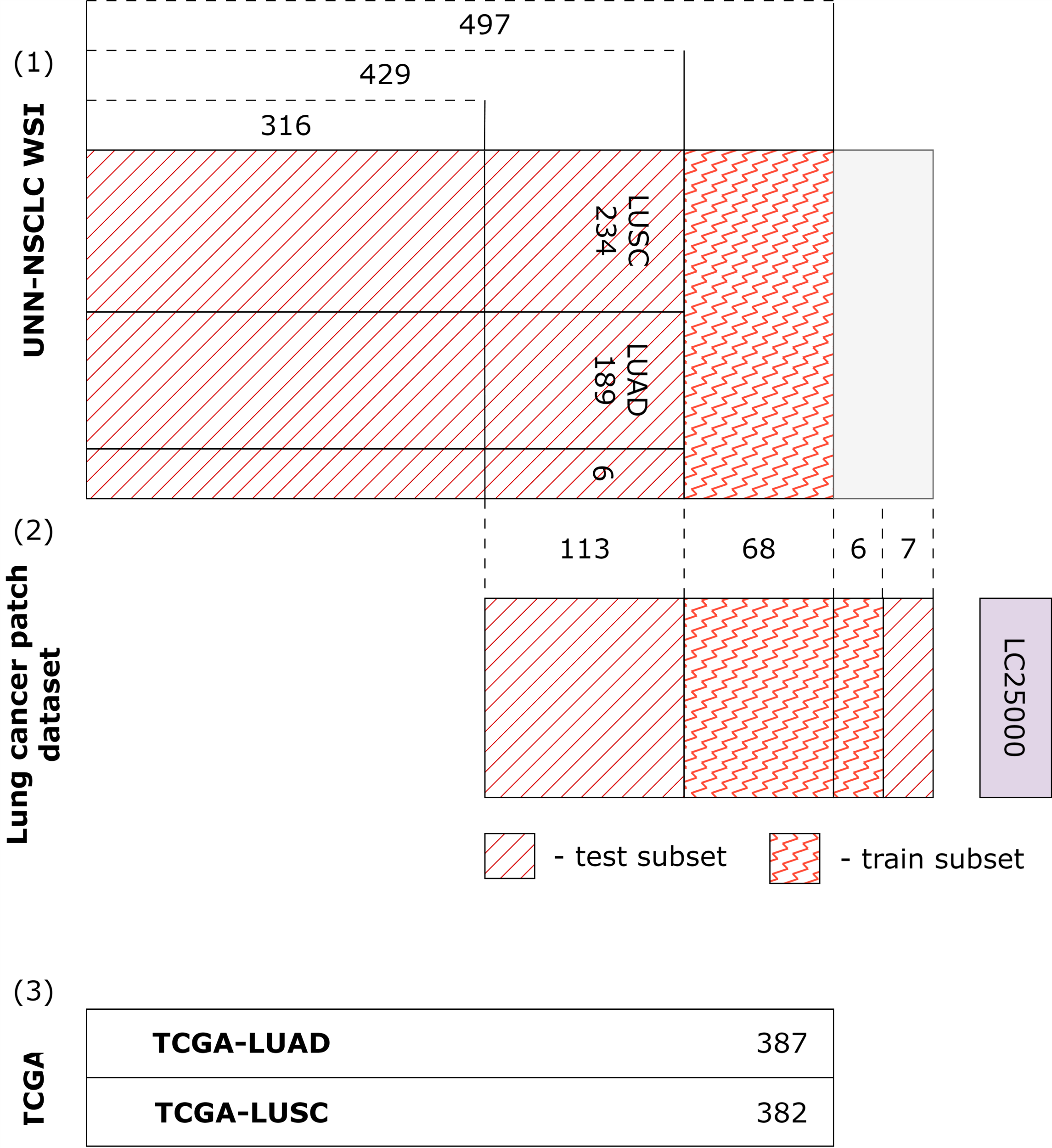


Figure A1.1 Distribution and relations of WSIs used in training and testing of the pipeline for (1) UNN-NSCLC WSI dataset, (2) Lung cancer patch dataset and (3) TCGA dataset

## Supplementary Results


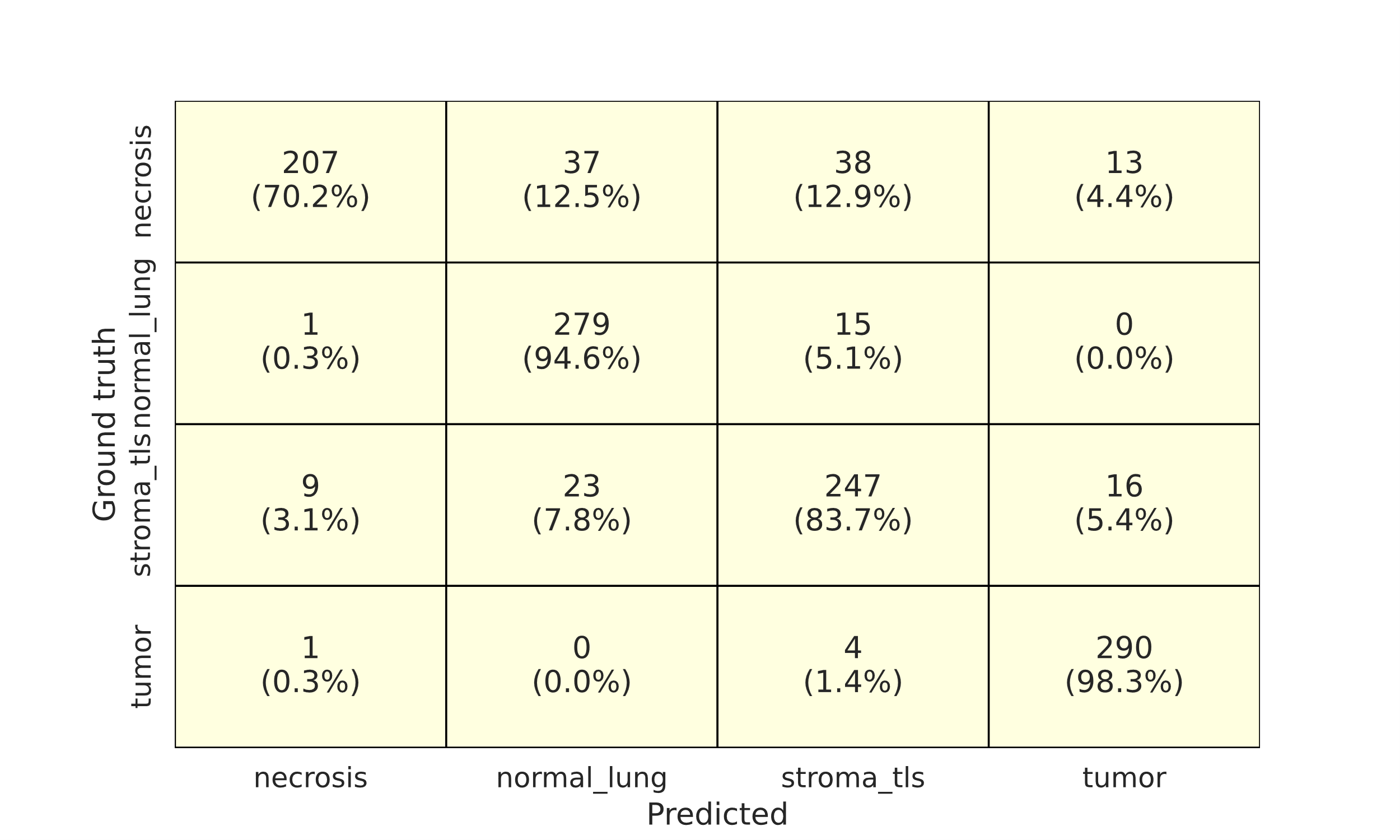


Figure A2.1 Confusion matrix of the classification model for the test subset

| **Patch ratio** | **c-index (average)** | **Standard deviation** | **Number of patches (average)** |
| --- | --- | --- | --- |
| 0.005 | 0.63214 | 0.0113 | 21 |
| 0.01 | 0.63904 | 0.0083 | 42 |
| 0.05 | 0.64851 | 0.0035 | 215 |
| 0.1 | 0.65090 | 0.0023 | 432 |
| 0.2 | 0.65144 | 0.0017 | 864 |
| 0.3 | 0.65177 | 0.0014 | 1297 |
| 0.4 | 0.65209 | 0.0010 | 1730 |
| 0.5 | 0.65190 | 0.0009 | 2163 |

Table A2.1 Analyzed patch ratios and corresponding c-index calculated using Monte-Carlo simulation and 100 iterations

## Supplementary Validation

### Additional TILs score analysis

In our additional analyses, we examine the relationships between the TILs score and clinicopathological variables to assess potential confounding factors and validate the independence of the TILs score as a prognostic marker (Appendix Table A3.2). We use chi-squared tests when the expected counts are sufficiently large (E_c_ >= 5) and apply Fisher's exact tests in other cases, to identify any significant associations. In the test subset (n = 429), our analyses reveal no significant associations between the TILs score and key clinicopathological variables such as age, gender, weight loss, smoking status, ECOG performance status, pStage, differentiation, and vascular invasion (p > 0.05). The high p-values suggest that the TILs score is independent of these potential confounders and can potentially be an unbiased prognostic factor.

Further exploring the biological value of the TILs score, we examine its relationship with the expression levels of IHC-based markers of common immune cells: CD3 (pan T-cells), CD4 (helper T-cells), CD8 (cytotoxic T-cells), and CD20 (B-cells), as presented in Appendix Table A3.3. Patients in the highest TILs score quartile (Q4) have a significantly greater proportion of high CD8 expression compared to those in the lowest quartile (Q1). Cochran-Armitage tests for trend are conducted to assess the presence of trends in TILs score quartiles and the expression levels of the immune cell markers. The test results demonstrate significant positive trends for all markers, with the most pronounced observed for CD3 (Z = 7.16, p < 0.001) and the least for CD20 (Z = 3.43, p < 0.001). These findings suggest that the TILs score, as a measure of immune cell infiltration, exhibits a monotonously growing trend across clinical markers from Q1 to Q4.

To evaluate the prognostic value of the TILs score in a univariate model, we examine both the TILs score and the CD8 IHC score separately for predicting disease-specific survival in NSCLC patients, as detailed in Appendix Table A3.4. In the test subset, patients in the highest TILs score quartile (Q4) have a significantly reduced hazard of disease-specific death compared to those in the lowest quartile (Q1), with HR of 0.26 (95% CI: 0.16–0.40). The CD8 IHC score also shows a trend towards improved survival in higher quartiles, though the hazard is higher for Q4 with HR of 0.46 (95% CI: 0.30–0.70).

We also perform Kaplan-Meier survival analyses stratified on histology (Appendix Figure A3.1) and pStage (Appendix Figure A3.2). In histologically differentiated patients, Kaplan-Meier survival analyses demonstrate a distinct stratification between the lowest and highest quartiles of TILs scores in both LUAD and LUSC. For pStage, we observe a consistent trend where higher TILs scores are linked to better survival outcomes across all stages. The most pronounced separation between risk groups is observed in early-stage tumors.

|  | Test subset (LUSC and LUAD) | | LUSC subset | | LUAD subset | |
| --- | --- | --- | --- | --- | --- | --- |
|  | Baseline | Extended | Baseline | Extended | Baseline | Extended |
| likelihood ratio | -903.80 | -891.72 | -358.31 | -352.31 | -411.72 | -408.09 |
| χ^2^ | 24.159 | | 12.006 | | 7.2584 | |
| p | 8.87×10^-7^ | | 0.0005304 | | 0.007057 | |

Table A3.1 Likelihood Ratio Test results comparing Baseline (pStage + Differentiation) and Extended (pStage + Differentiation + TILs) cox regression models

|  | All patients (n=497) | | | | | Test patients (n=429) | | | | | Train patients (n=68) | | | | |
| --- | --- | --- | --- | --- | --- | --- | --- | --- | --- | --- | --- | --- | --- | --- | --- |
|  | TILs score | | | | | TILs score | | | | | TILs score | | | | |
|  | Q1 | Q2 | Q3 | Q4 | p | Q1 | Q2 | Q3 | Q4 | p | Q1 | Q2 | Q3 | Q4 | p |
| Age |  | | | | | | | | | | | | | | |
| <65 | 50 | 57 | 47 | 53 | 0.649 | 47 | 45 | 41 | 45 | 0.880 | 3 | 12 | 6 | 8 | 0.393 |
| ≥65 | 62 | 76 | 80 | 72 |  | 61 | 62 | 66 | 62 |  | 1 | 14 | 14 | 10 |  |
| Gender |  | | | | | | | | | | | | | | |
| Female | 31 | 41 | 43 | 45 | 0.542 | 30 | 37 | 36 | 41 | 0.431 | 1 | 4 | 7 | 4 | 0.441 |
| Male | 81 | 92 | 84 | 80 |  | 78 | 70 | 71 | 66 |  | 3 | 22 | 13 | 14 |  |
| Weight loss |  | | | | | | | | | | | | | | |
| <10% | 103 | 11 | 110 | 117 | 0.268 | 99 | 97 | 90 | 101 | 0.118 | 4 | 20 | 20 | 16 | 0.094 |
| ≥10% | 9 | 16 | 16 | 8 |  | 9 | 10 | 16 | 6 |  | 0 | 6 | 0 | 2 |  |
| Smoking status |  | | | | | | | | | | | | | | |
| Never smoked | 5 | 4 | 1 | 7 | 0.113 | 5 | 2 | 0 | 6 | 0.106 | 0 | 2 | 1 | 1 | 0.294 |
| Present smoker | 64 | 80 | 89 | 83 |  | 63 | 66 | 75 | 69 |  | 1 | 14 | 14 | 14 |  |
| Previous smoker | 43 | 49 | 37 | 35 |  | 40 | 39 | 32 | 32 |  | 3 | 10 | 5 | 3 |  |
| ECOG status |  | | | | | | | | | | | | | | |
| Normal | 57 | 76 | 77 | 84 | 0.110 | 55 | 66 | 65 | 70 | 0.284 | 2 | 10 | 12 | 14 | 0.124 |
| Slightly reduced | 42 | 47 | 43 | 37 |  | 41 | 35 | 36 | 33 |  | 1 | 12 | 7 | 4 |  |
| In bed <50% | 13 | 10 | 7 | 4 |  | 12 | 6 | 6 | 4 |  | 1 | 4 | 1 | 0 |  |
| Histology |  | | | | | | | | | | | | | | |
| LUSC | 62 | 70 | 63 | 82 | 0.085 | 59 | 56 | 51 | 68 | 0.129 | 3 | 14 | 12 | 14 | 0.619 |
| LUAD | 50 | 61 | 61 | 41 |  | 49 | 50 | 53 | 37 |  | 1 | 11 | 8 | 4 |  |
| Other | 0 | 2 | 3 | 2 |  | 0 | 1 | 3 | 2 |  | 0 | 1 | 0 | 0 |  |
| pStage |  | | | | | | | | | | | | | | |
| IA | 26 | 33 | 33 | 41 | 0.117 | 25 | 27 | 30 | 35 | 0.157 | 1 | 6 | 3 | 6 | 0.875 |
| IB | 17 | 20 | 29 | 12 |  | 16 | 15 | 24 | 11 |  | 1 | 5 | 5 | 1 |  |
| IIA | 8 | 11 | 14 | 12 |  | 7 | 8 | 11 | 10 |  | 1 | 3 | 3 | 2 |  |
| IIB | 25 | 32 | 25 | 37 |  | 25 | 24 | 19 | 32 |  | 0 | 8 | 6 | 5 |  |
| IIIA | 32 | 31 | 20 | 20 |  | 31 | 27 | 17 | 16 |  | 1 | 4 | 3 | 4 |  |
| IIIB | 4 | 6 | 6 | 3 |  | 4 | 6 | 6 | 3 |  | 0 | 0 | 0 | 0 |  |
| Differentiation |  | | | | | | | | | | | | | | |
| Poor | 47 | 51 | 58 | 50 | 0.693 | 46 | 42 | 49 | 42 | 0.872 | 1 | 9 | 9 | 8 | 0.635 |
| Moderate | 52 | 61 | 53 | 52 |  | 49 | 48 | 43 | 46 |  | 3 | 13 | 10 | 6 |  |
| Well | 13 | 21 | 16 | 23 |  | 13 | 17 | 15 | 19 |  | 0 | 4 | 1 | 4 |  |
| Vascular invasion |  | | | | | | | | | | | | | | |
| No | 91 | 113 | 102 | 104 | 0.725 | 88 | 90 | 88 | 88 | 0.930 | 3 | 23 | 14 | 16 | 0.322 |
| Yes | 21 | 19 | 24 | 20 |  | 20 | 16 | 18 | 18 |  | 1 | 3 | 6 | 2 |  |

Table A3.2 Quantized TILs score distribution with clinicopathological variables for UNN-NSCLC WSI dataset. Patient numbers depend on availability of clinical data from the respective subset

|  | All patients (n=497) | | | | | | Test patients (n=429) | | | | | | Train patients (n=68) | | | | | |
| --- | --- | --- | --- | --- | --- | --- | --- | --- | --- | --- | --- | --- | --- | --- | --- | --- | --- | --- |
|  | TILs score | | | | | | TILs score | | | | | | TILs score | | | | | |
|  | Q1 | Q2 | Q3 | Q4 | z | p | Q1 | Q2 | Q3 | Q4 | z | p | Q1 | Q2 | Q3 | Q4 | z | p |
| CD3 |  | | | | | | | | | | | | | | | | | |
| [0,1e+03] | 103 | 109 | 89 | 60 | 7.68 | <0.001 | 99 | 90 | 73 | 54 | 7.16 | <0.001 | 4 | 19 | 16 | 6 | 2.76 | <0.05 |
| (1e+03,5e+03] | 9 | 23 | 37 | 61 |  |  | 9 | 17 | 33 | 51 |  |  | 0 | 6 | 4 | 10 |  |  |
| CD4 |  | | | | | | | | | | | | | | | | | |
| [0,550] | 90 | 86 | 65 | 57 | 5.98 | <0.001 | 87 | 72 | 54 | 53 | 5.34 | <0.001 | 3 | 14 | 11 | 4 | 2.48 | <0.05 |
| (550,5e+03] | 21 | 44 | 60 | 68 |  |  | 20 | 34 | 51 | 54 |  |  | 1 | 10 | 9 | 14 |  |  |
| CD8 |  | | | | | | | | | | | | | | | | | |
| [0,500] | 76 | 64 | 56 | 36 | 5.88 | <0.001 | 73 | 53 | 44 | 31 | 5.77 | <0.001 | 3 | 11 | 12 | 5 | 1.26 | 0.21 |
| (500,5e+03] | 36 | 69 | 71 | 89 |  |  | 35 | 54 | 63 | 76 |  |  | 1 | 15 | 8 | 13 |  |  |
| CD20 |  | | | | | | | | | | | | | | | | | |
| [0,400] | 101 | 109 | 100 | 92 | 3.46 | <0.001 | 97 | 88 | 84 | 78 | 3.42 | <0.001 | 4 | 21 | 16 | 14 | 0.70 | 0.48 |
| (400,5e+03] | 10 | 23 | 27 | 33 |  |  | 10 | 18 | 23 | 29 |  |  | 0 | 5 | 4 | 4 |  |  |

Table A3.3 Quantized TILs score distribution over IHC based immune cell marker subsets and Cochran-Armitage test results for trend (z)

|  | All patients (n=497) | | | | | Test patients (n=429) | | | | | Train patients (n=68) | | | | |
| --- | --- | --- | --- | --- | --- | --- | --- | --- | --- | --- | --- | --- | --- | --- | --- |
|  | N (%) | 5 Year | Median | HR (95%CI) | p | N (%) | 5 Year | Median | HR (95%CI) | p | N (%) | 5 Year | Median | HR (95%CI) | p |
| CD8_posmm2_median | | | | | | | | | | | | | | | |
| Q1 | 124 (25) | 45 | 42 | 1 | <0.001 | 108 (25) | 45 | 45 | 1 | <0.001 | 16 (24) | 40 | 25 | 1 | 0.08 |
| Q2 | 123 (25) | 52 | 73 | 0.82 (0.54-1.25) |  | 107 (25) | 50 | 68 | 0.88 (0.57-1.38) |  | 16 (24) | 61 | NR | 0.47 (0.13-1.66) |  |
| Q3 | 126 (25) | 71 | 235 | 0.45 (0.3-0.68) |  | 107 (25) | 68 | NR | 0.49 (0.32-0.76) |  | 19 (28) | 88 | 235 | 0.24 (0.07-0.82) |  |
| Q4 | 124 (25) | 70 | NR | 0.5 (0.33-0.75) |  | 107 (25) | 72 | NR | 0.46 (0.3-0.7) |  | 17 (25) | 62 | 84 | 0.73 (0.2-2.58) |  |
| TILs score | | | | | | | | | | | | | | | |
| Q1 | 112 (23) | 36 | 32 | 1 | <0.001 | 108 (25) | 37 | 32 | 1 | <0.001 | 4 (6) | 33 | 30 | 1 | 0.12 |
| Q2 | 133 (27) | 54 | 75 | 0.62 (0.4-0.96) |  | 107 (25) | 54 | 98 | 0.61 (0.38-0.96) |  | 26 (38) | 53 | 61 | 0.75 (0.09-6.41) |  |
| Q3 | 127 (26) | 67 | NR | 0.41 (0.27-0.64) |  | 107 (25) | 65 | NR | 0.45 (0.28-0.71) |  | 20 (29) | 78 | NR | 0.28 (0.03-2.39) |  |
| Q4 | 125 (25) | 78 | 235 | 0.27 (0.18-0.42) |  | 107 (25) | 79 | NR | 0.26 (0.16-0.4) |  | 18 (26) | 72 | 235 | 0.35 (0.04-2.96) |  |

Table A3.4 Quantized CD8 TILs density and TILs score as predictors of disease-specific survival in non-small cell lung cancer patients. HR and p-values are calculated based on 5-year survival. Abbreviations: NR, not reached


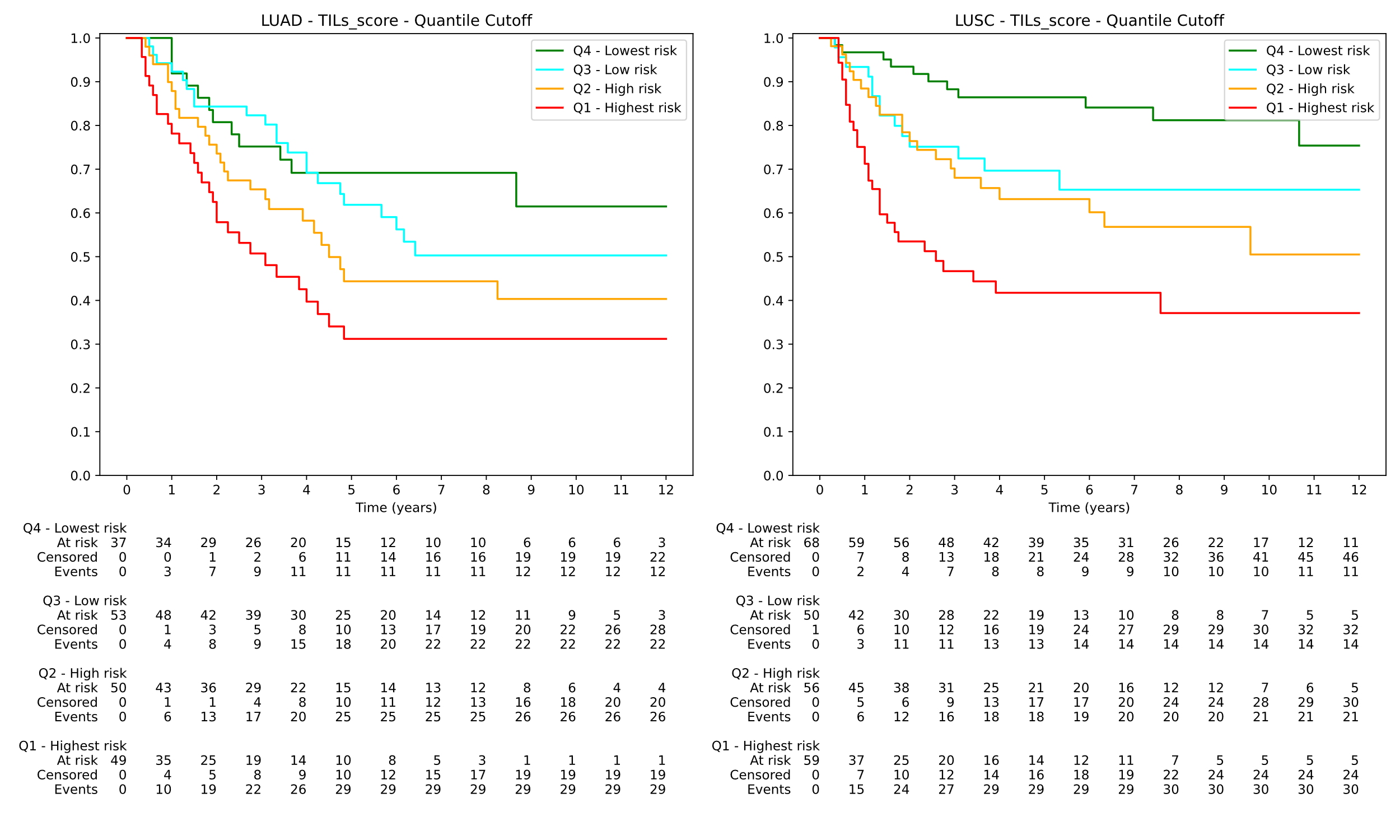


Figure A3.1 Kaplan-Meier curves for TILs score stratified by histology


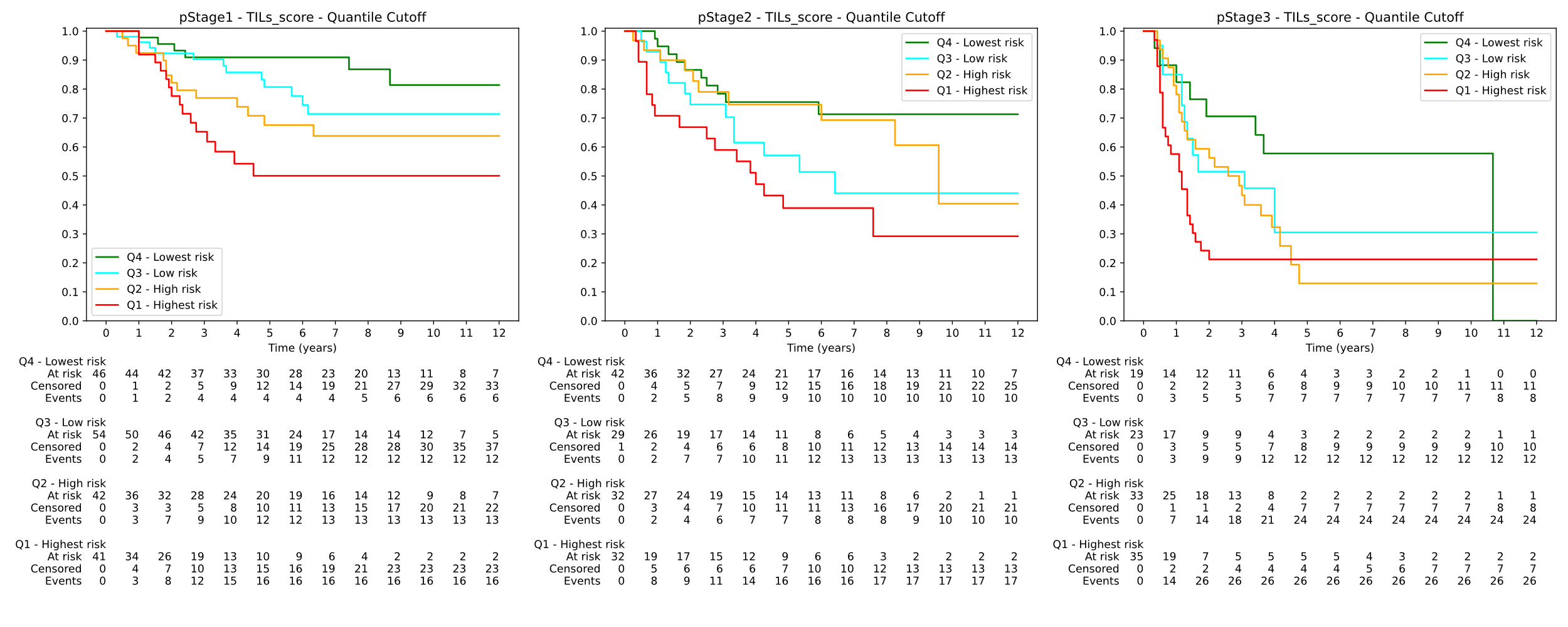


Figure A3.2 Kaplan-Meier curves for TILs score stratified by pStage
